# PD-L1 in Cytological Samples: A Review and a Practical Approach

**DOI:** 10.3389/fmed.2021.668612

**Published:** 2021-05-07

**Authors:** Eva Tejerina, Laura Garca Tobar, Jos I. Echeveste, Carlos E. de Andrea, Elena Vigliar, Mara D. Lozano

**Affiliations:** ^1^Department of Pathology, Hospital Universitario Puerta de Hierro, Madrid, Spain; ^2^Department of Pathology, Clinica University of Navarra, Pamplona, Spain; ^3^Department of Public Health, University of Naples Federico II, Naples, Italy

**Keywords:** non-small cell lung cancer, cytopathology, PD-L1, immunocytochemistry, molecular cytopathology

## Abstract

With a growing number of predictive biomarkers needed to manage patients with non-small cell lung cancer (NSCLC), there has been a paradigm shift in care and handling of diagnostic samples. Among the various testing methods, immunohistochemistry (IHC) is the most cost- effective and widely available. Furthermore, over the past decade immunotherapy has emerged as one of the most promising cancer treatments. In this scenario IHC is the most used testing method available for PDL-1/PD1 immunotherapy. Several monoclonal antibodies targeting programmed death 1 (PD-1)/programmed death ligand-1 (PD-L1) pathways have been integrated into standard-of-care treatments of a wide range of cancer types, once provided evidence of PD-L1 expression in tumor cells by immunohistochemistry (IHC). Since currently available PD-L1 assays have been developed on formalin-fixed paraffin embedded (FFPE) histological specimens, a growing body of research is being dedicated to confirm the feasibility of applying PDL-1 assays also to cytological samples. Albeit promising results have been reported, several important issues still need to be addressed. Among these are the type of cytological samples, pre-analytical issues, cyto-histological correlation, and inter-observer agreement. This review briefly summarizes the knowledge of the role of cytopathology in the analysis of PD-L1 by immunocytochemistry (ICC) and future directions of cytopathology in the immunotherapy setting.

## Introduction

Immunotherapy, particularly the clinical development of immune-checkpoint inhibitors (ICIs), has emerged as one of the most promising cancer treatments. Programmed death cell ligand-1 (PD-L1) immunohistochemical expression has been integrated into standard-of-care treatment of NSCLC (1). In this scenario the expression of PD-L1 by immunohistochemistry (IHC), although an imperfect marker, is the most widely used testing method for treatment recommendations with ICIs. It is well-known that so far ICIs therapies work only for a subset of patients and, although IHQ expression of PD-L1 is used for treatment recommendations of certain ICIs, not all patients whose tumors show high expression of PD-L1 will benefit from these drugs.

Several monoclonal antibodies targeting PD-1/PD-L1 pathway have been integrated into standard-of-care treatments in NSCLC, provided evidence of PD-L1 expression in tumor cells by IHC. Currently, all commercially available immunohistochemistry assays have been validated to be used with FFPE specimens (2,4). However, in routine clinical practice about 4050% of NSCLC patients have only cytology samples available for diagnosis, staging, and biomarker analysis (5). Consequently, pathologists, mainly those dedicated to cytopathology, have no choice but to resort, implement, validate, and take advantages of cytological specimens for diagnosis and biomarker analysis. In this setting molecular cytopathology has become a significant player in the world of diagnosis and predictive pathology and a growing body of research is being dedicated to validate the feasibility of applying PDL-1 assays to cytological samples (5,19).

Several studies have explored the issue of using cytology as an alternative to surgical specimens for PD-L1 testing. Many of them have advocated that the samples obtained by endobronchial ultrasound-guided transbrochial needle aspiration (EBUS-TBNA/TBNB) or endoscopic ultrasound-guided needle aspiration (FNA EUS-FNA/FNAB) are as suitable as surgical specimens to test PD-L1 (4, 7,13). Albeit promising results have been reported, several important issues still need to be addressed. Among these are the type of cytological samples, pre-analytical issues, validation studies and controls, cyto-histological correlation, centralized vs. in house testing, and inter-observer agreement.

## Type of Cytological Samples and Preanalitics

The rationale of cytopathology is to provide adequate cellular material in order not only to make an accurate diagnosis and staging, but also to perform ancillary tests with prognostic and/or therapeutic value necessary for the adequate clinical management of NSCLC patients. EBUS-TBNA/TBNB, specifically developed to collect samples from the lung and/or mediastinum, is one of the most commonly procedures performed in this context. Together with EUS-FNA/FNAB these techniques allow for minimally invasive diagnosis and staging of lung and mediastinal lesions (20,24).

The success of this procedure relies on an accurate specimen collection and handling, appropriate triage of the samples, and processing (25). Rapid on-site Evaluation (ROSE) by a cytopathologist or a trained cytotechnologist is recommended. ROSE allows for an adequate triage of the sample for diagnosis and biomarker analysis and increases diagnostic accuracy maintaining an exhaustive pre-analytical control, among many other advantages.

Different types of cytological samples are used in routine practice. The International Association for the Study of Lung Cancer (IASLC) Pathology committee states that all cytologic preparations, including cell blocks, ethanol fixed, and air-dried slides can be used for immunocytochemistry (ICC) (26). However, since each type is characterized by its particular preanalytical issues, each one should be considered as a separate entity. Table 1 shows some peculiarities of the different types of cytological samples to be taken into account. Specific recommendations for PD-L1 testing in cytology are yet to be established and validated (27).

**Table 1:** Peculiarities and performance of different types of cytological samples for ICC analysis.

**Type of cytological samples**	**Fixative**	Results*
Cellblock	Formalin	Comparable results to surgical samples
Papanicolaou-stained smears	Alcohol 96	Comparable results to surgical samples
Unstained smears	Alcohol 96	Slightly lower but OK
DQ and air dried smears	No fixative	High rate of false negatives low intensity of immunostaining
Liquid based	Metanol-based fixatives	High rate of false negatives low intensity of immunostaining

, *rigorous validation and protocol optimization should be performed in each laboratory that performs IHC on cytology specimens (e.g., alcohol-fixed cell blocks, air-dried smears, formalin-post-fixed specimens).

Furthermore, PD-L1 ICC in cytological samples has not even considered as an alternative to FFPE in many institutions. Cell blocks are the most widely used due to the same management as other FFPE specimens. However, not all cell blocks are prepared in the same way, and furthermore some of them are not very cellular, hence the need to optimize other types of cytological samples for PD-L1 analysis (14, 27). The main recommendation is to fix the cell block in 10% buffered formalin, although some authors (10, 28, 29) have shown that the type of fixative does not affect PD-L1 staining. In this sense, our group have demonstrated a good concordance in PD-L1 expression between FFPE samples, FFPE cell blocks, and alcohol-fixed Papanicolaou stained smears (14).

Non-cell block cytological preparations (air-dried and alcohol-fixed direct smears, cytospins, and liquid-based cytology preparations) pose an even greater challenge for ICC validation. In our experience, among all these types of cytological samples we obtain much better results using alcohol-fixed, Papanicolau-stained smears (14) (Table 1). Papanicolaou stain helps to identify areas of interest and, according to the manufacturer's specifications, do not require to be previously destained (14). Rapid-H&E destaining previously to immunohistochemistry is an alternative procedure (6). Lozano et al. recommend using coverslipping film instead of a glass coverslip in order to shorten coverslip removal time and avoid hypothetical cell losses when removing crystal coverslip for performing ICC (14).

While there are studies in the literature suggesting that some fixatives other than formalin can alter the antigenicity and then results of ICC in cytological samples, a report from the United Kingdom National External Quality Assessment Service (UK NEQAS) indicates that all non- formalin fixatives, apart from acetone, yield a comparable quality of immunostaining than formalin (30).

**Figure 1:**
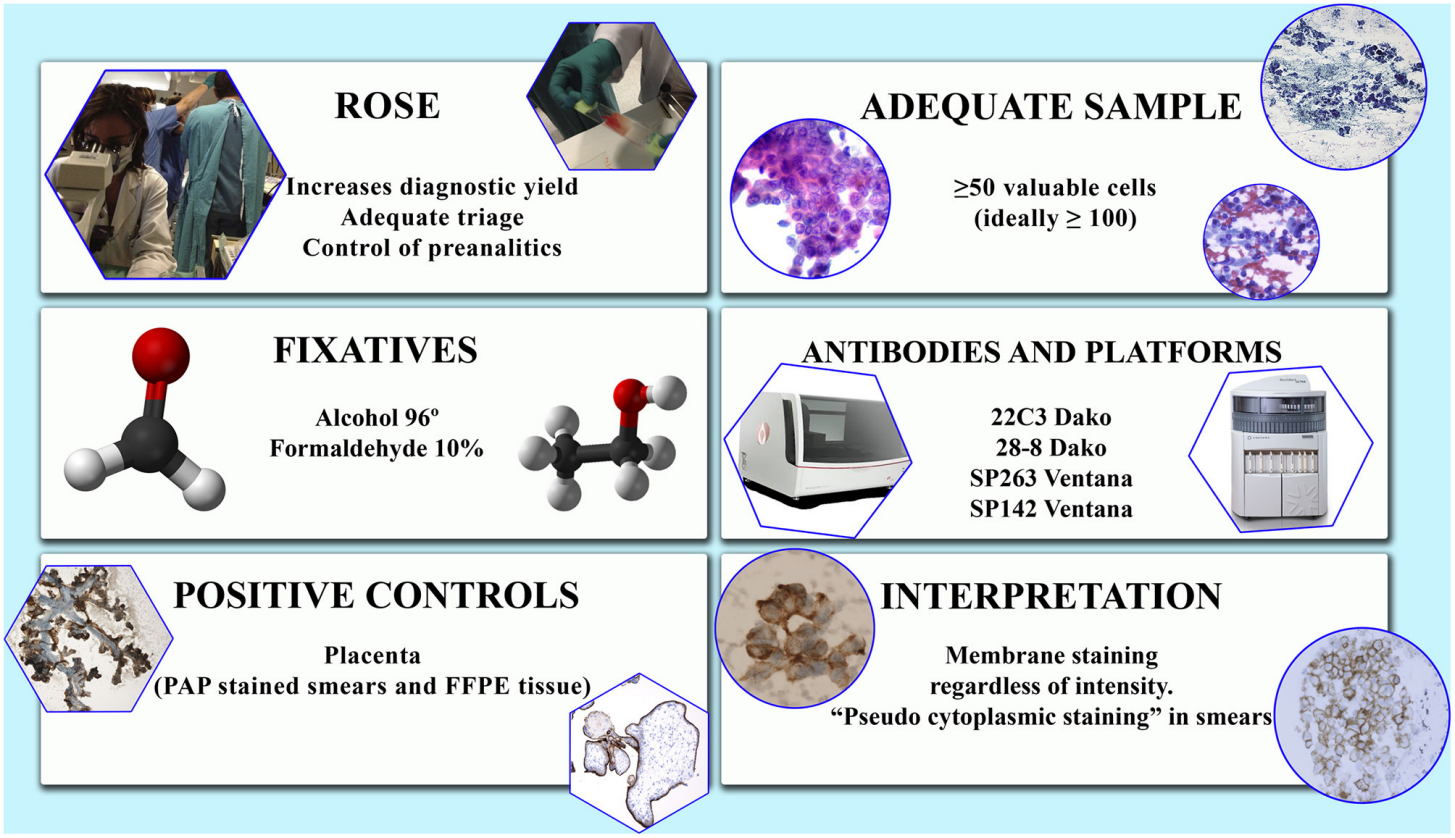
Some issues to be considered in the study of PD-L1 in cytological samples. Factors to be evaluated.

The use of positive and negative controls is mandatory. Placental tissue (even in the form of Papanicolaou-stained smears) as well as macrophages can be used as external and internal positive controls, respectively (4, 14) (Figure 1).

## Microscopic Criteria of Adequacy and Scoring

Although the criteria of adequacy for cytological samples are still to be approved and validated, most studies recommend analyzing 50 well-preserved cells (1). In a study from our group comparable results among smears, cell blocks and resected specimens were obtained analyzing at least 100 viable, well-preserved, not overlapped cells (14). The same criteria of cellularity were used by others (15). ROSE can improve specimen quality in terms of tumor cellularity (14, 16).

Due to the existence of different assays, each of which includes their own antibody and staining platform, there is a considerable heterogeneity in the evaluation criteria of the PD-L1 immunohistochemical tests. This heterogeneity is observed not only in the patterns and intensity of staining but also, and more importantly, in the score system and in the cut-off levels that determine if a sample is positive or not (31) (Figure 1).

Some authors advocate the use in cytology of scoring systems and cut-offs similar to those used in histology to assess PD-L1 expression (7). However, this issue probably needs to be more deeply explored. Thus, overall concordance rates vary depending on the positivity cut-off point used in the different studies. Using a 3-tiered score system of TPS<1% for negative cases, TPS149%, for positive cases and TPS50% for positive cases with high PD-L1 expression, the global concordance rate between paired cytological smears and FFPE (that included cell- blocks, small biopsies and surgical specimens) was as high of 97.3% (*p* < 0.05) (14). Munari et al. reported a global concordance rate of 90.6% by using a two-tiered score of <50% and 50% TPS for negative and positive cases, respectively; the overall agreement decreased to 81.3% when using a 1% cut-off (15). Kuempers et al. reported the lowest global concordance rate (53%) by evaluating paired samples according to continuous values of expression, that raised to 74.1% when PD-L1 expression was categorized based on TPS and to 82% when a deviation of PD-L1 expression of 10% was admitted (17).

To our knowledge the discrepancies depending on whether one or other assay is performed in cytological material are not statistically significant (14). Nevertheless, as previously mentioned, more studies are needed to establish the most accurate evaluation of PD-L1 in cytological samples. The feasibility of the evaluation of PD-L1 expression in tumor-associated inflammatory cells (TILs) is of little value at present time and is not being considered, except as an internal control.

Rigorous validation and protocol optimization should be performed in each laboratory that performs IHC on cytology specimens (e.g., alcohol-fixed cell blocks, air-dried smears, formalin post-fixed specimens) (14, 18, 19, 26, 30, 32,35).

The distinction between inflammatory cells (mainly macrophages) and tumor cells in the analytical phase is essential for an accurate evaluation of PD-L1 status. As previously mentioned, their differentiation, even more after immunocytochemical staining, can be challenging in cytological samples. Although the PD-L1 score is independent of the intensity of the staining, differences between assays can add more difficulties in the evaluation of the test (14, 36). Vigliar et al. reported a lower intensity of staining with the Dako platform when compared with the Ventana platform that interestingly made the distinction between macrophages and neoplastic cells easier with the first (36). This issue could also be avoided if the smear selected for the assay is scanned prior to performing ICC in order to properly identify the tumor cells (15). Scanning of slides will become routine practice if PD-L1 scoring using digital images is finally validated (13).

## Cyto-Histologic Correlation. Intra and Inter-Observer Reproducibility

To establish whether cytological samples are as reliable as the histological samples for PD-L1 testing, numerous authors have intensively investigated the concordance rates between matched cytological and histological samples. Since 2017 several single institutional studies have reported comparable PD-L1 expression on matched cytological and histological (small biopsy/surgical resection) specimens (7, 8, 10, 11, 14, 37,44).

Regarding cytological samples, Kuempers et al. have reported a significantly higher interobserver variability in the assessment of PD-L1 expression for cytology when evaluating paired cyto-histological samples (17). However, Munari et al. reported an excellent intraobserver agreement and a good interobserver concordance with SP263 assay (98.1 and 90.5%, respectively) (15). More studies are needed to establish the influence of clones and cut-off points in cytological material.

## Interpretation and Reporting

The objective of PD-L1 testing is to select those patients with NSCLC who are likely to benefit from immunotherapy with ICIs. Cytological samples have some intrinsic characteristics that can add more difficulties to the evaluation of the tests. The need to identify true tumor cells among normal and/or inflammatory cells (mainly macrophages, which are positive for PD-L1 antibodies and therefore can be used as positive internal controls) and to select well-preserved, not overlapped cells, makes recommendable to allocate the evaluation of the test to an experienced cytopathologist (14, 16).

Likewise to histological specimens, any intensity of linear membrane staining, partial or complete, is considered positive also in cytologic samples (2) (Figure 1). Focal nuclear and cytoplasmic granular staining is considered artifactual, as well as diffuse, exclusively cytoplasmic staining (14, 15). Specimens with intense non-specific background staining should not be evaluated (14). Some peculiarities in PD-L1 immunoreactivity in cytological specimens have been noticed: (1) a folded, thick and strong membranous staining due to the three- dimensionality of the cells in the smears; (2) a light submembranous cytoplasmic reinforcement; and (3) a rare perinuclear dot-like staining when using 22C3 assay (14) (Figure 1). The presence of light cytoplasmic staining in addition to membranous has been observed by others (15). As in histological material, staining heterogeneity among different samples of a same tumor or within a single preparation has been also observed (14).

Following the recommendations of the International Association for the Study of Lung Cancer (IASLC) and analogously to histological specimens, tumor proportion score (TPS) should be used to report the result of the PD-L1 immunohistochemical tests performed on cytological samples (14, 15, 17). The type of sample, type of assay (including the clone and the platform) and the scoring system should also be included in the report.

## Intra and Inter-Observer Reproducibility

The intra- and interobserver discrepancies in the evaluation of PD-L1 by IHC have been mostly evaluated in histological specimens, with different results depending on the clone and the cut- off point selected. Cooper et al. found that the interobserver concordance was lower when using a 50% cut-off than with a 1% TPS (81.9 and 84.2%, respectively), but the opposite happened with the intraobserver agreement (91.3 and 89.7%, respectively) (45). Brunnstrm et al. reported an overall interobserver agreement of 0.710.96 kappa values; nevertheless, it was significantly better when using a 50% cut-off point than with a 1% TPS (46). Training sessions prior to evaluation does not seem to affect the results (37). The possible influence of the clone used for immunohistochemistry has also been explored. Some authors found similar results in terms of interobserver reproducibility regardless the test performed, while others describe the highest concordance when using 22C3 and 28-8 and the lowest with SP142 (12, 46).

Regarding cytological samples, Kuempers et al. have reported a significantly higher interobserver variability in the assessment of PD-L1 expression for cytology when evaluating paired cyto-histological samples (17). However, Munari et al. reported an excellent intraobserver agreement and a good interobserver concordance with SP263 assay (98.1 and 90.5%, respectively) (15). More studies are needed to establish the interobserver concordance. Likely, the implementation of algorithms for digital scoring will help.

## Future Perspectives and Conclusions

Nowadays a new scenario is opening in the field of immunotherapy. It is widely known that predicting ICIs therapy outcomes based solely on PD-L1 is far from perfect. Therefore, promising predictive biomarkers are currently under investigation. One of those is TMB, defined as the total number of somatic mutations per tumor genome. Although almost all the data on TMB derive from evaluation of FFPE histological samples, preliminary results on the feasibility of assessing TMB on cytological material have been already published (47, 48).

Another promising line of research is the study of the tumor microenvironment using multiplexed techniques, mainly multiplex immunohistochemistry/immunofluorescence (mIHC/IF). This highly-throughput technique allows the detection of multiple markers on a single slide, providing simultaneously standardized quantitative analysis of the results and accordingly avoiding interobserver variability (49, 50). The procedure allows therefore to explore not only a single cell population but also the cellular composition and the relationship between different immune cell types and/or immune cells and tumor cells in different fields. A recent study (51) has characterized different types of immune cells in human tuberculosis granulomas using ultra-fast cycling for multiplexed cellular fluorescence imaging. This supports the promising usefulness of the procedure to explore the role of the tumor microenvironment (TME) and its dynamic changes related to cancer progression and/or the effect of treatment.

Although developed on FFPE material, its feasibility on cytological cell blocks and small samples has been recently reported (52). This increases the value of cytology as a substrate to test new biomarkers and strengthens its role in the management of cancer patients.

In conclusion, with the increasing number of predictive biomarkers available for the management of NSCLC patients, the need to improve rapid, reliable, standardized, reproducible, and cost-effective results from minimally invasive samples from NSCLC patients is critical. PD-L1 assessment in cytological samples, although certainly poses some challenges, has proven to be useful, efficient, safe and reliable in experienced hands. The data available to date clearly indicate that, with proper optimization and rigorous quality controls and internal and external validation, PD-L1 ICC can be performed successfully on cytological specimens.

## Author Contributions

ML: conceptualization, methodology, writing, review, editing, and supervision. ET: writing and original draft preparation. ML and JE: figure design. All authors validation, investigation, and visualization.

## Conflict of Interest

The authors declare that the research was conducted in the absence of any commercial or financial relationships that could be construed as a potential conflict of interest.
